# VAMP5 and distinct sets of cognate Q-SNAREs mediate exosome release

**DOI:** 10.1247/csf.23067

**Published:** 2023-09-14

**Authors:** Takahide Matsui, Yuriko Sakamaki, Shu Hiragi, Mitsunori Fukuda

**Affiliations:** 1 Laboratory of Membrane Trafficking Mechanisms, Department of Integrative Life Sciences, Graduate School of Life Sciences, Tohoku University, Aobayama, Aoba-ku, Sendai, Miyagi 980-8578, Japan; 2 Department of Molecular Oncology, Institute for Advanced Medical Sciences, Nippon Medical School, Tokyo 113-8602, Japan; 3 Microscopy Research Support Unit Research Core, Tokyo Medical and Dental University, Tokyo 113-8510, Japan

**Keywords:** exosome, small extracellular vesicle (sEV), multivesicular body, SNARE, VAMP5

## Abstract

Small extracellular vesicles (sEVs) are largely classified into two types, plasma-membrane derived sEVs and endomembrane-derived sEVs. The latter type (referred to as exosomes herein) is originated from late endosomes or multivesicular bodies (MVBs). In order to release exosomes extracellularly, MVBs must fuse with the plasma membrane, not with lysosomes. In contrast to the mechanism responsible for MVB–lysosome fusion, the mechanism underlying the MVB–plasma membrane fusion is poorly understood. Here, we systematically analyze soluble *N*-ethylmaleimide-sensitive factor attachment protein receptor (SNARE) family proteins and identify VAMP5 as an MVB-localized SNARE protein required for exosome release. Depletion of VAMP5 in HeLa cells impairs exosome release. Mechanistically, VAMP5 mediates exosome release by interacting with SNAP47 and plasma membrane SNARE Syntaxin 1 (STX1) or STX4 to release exosomes. VAMP5 is also found to mediate asymmetric exosome release from polarized Madin-Darby canine kidney (MDCK) epithelial cells through interaction with the distinct sets of Q-SNAREs, suggesting that VAMP5 is a general exosome regulator in both polarized cells and non-polarized cells.

## Introduction

Exosomes are late endosome/multivesicular body (MVB)-derived membrane-wrapped nanoparticles that almost all types of cells release into the extracellular space. Although exosomes are now classified as endomembrane-derived sEVs, we referred them to exosomes herein to distinguish them from plasma-membrane-derived sEVs. Given exosomes carry specific bioactive molecules such as proteins, lipids, and nucleic acids, they are now recognized as a new mode of cell-cell communication, in which their cargos are transferred from a donor to a recipient cell via body fluids (Kalluri and LeBleu, 2020; Mathieu *et al.*, 2019; Pegtel and Gould, 2019; van Niel *et al.*, 2022). Exosomes have also been implicated in several pathologies, including cancers, neurodegenerative disorders, and infections (Kalluri and LeBleu, 2020; Pinnell *et al.*, 2021; Xu *et al.*, 2018).

In 1983, MVBs were first reported to fuse with the plasma membrane and that the fusion resulted in exosome release (Harding *et al.*, 1983; Pan and Johnstone, 1983). During the several decades since then three steps have been identified as being essential to exosome release. In the first step, intraluminal vesicles (ILVs), which contain future exosomal cargos, are formed by inward budding of the limiting membrane of MVBs into the lumen side, and in the second step, the secretory MVBs are specifically transported to the plasma membrane. In the third and final step, these MVBs fuse with the plasma membrane, which results in the release of the ILVs into the extracellular space as exosomes (Hessvik and Llorente, 2018; van Niel *et al.*, 2018; van Niel *et al.*, 2022). Since most of the MVBs are known to fuse with lysosomes, where their contents are degraded through the endocytic pathway (Huotari and Helenius, 2011; Yang and Wang, 2021), the secretory MVBs must selectively fuse with the plasma membrane, not with lysosomes, to release exosomes.

How the secretory MVBs fuse with the plasma membrane has never been fully elucidated. In general, specific membrane fusion events are achieved by soluble *N*-ethylmaleimide-sensitive factor attachment protein receptor (SNARE) complexes (Hong, 2005; Jahn and Scheller, 2006). During membrane fusion, a SNARE complex consisting of the Qa-, Qb-, Qc-, and R-SNAREs forms a parallel four-helix bundle with each SNARE contributing one helix to the bundle. Several individual SNARE proteins, including Syntaxin 5 (STX5; Qa-SNARE), YKT6 (R-SNARE), and VAMP7 (R-SNARE), have been reported to be involved in exosome release (Fader *et al.*, 2009; Gonzalez-Mendez *et al.*, 2020; Gross *et al.*, 2012; Hyenne *et al.*, 2015; Peak *et al.*, 2020; Verweij *et al.*, 2018; Wang *et al.*, 2021; Wei *et al.*, 2017). However, since YKT6 and STX5 are involved in endoplasmic reticulum (ER)-to-Golgi trafficking (Hardwick and Pelham, 1992; McNew *et al.*, 1997), their inhibition is likely to broadly affect other membrane fusion events. Moreover, although VAMP7, a well-known late endosomal and lysosomal R-SNARE (Advani *et al.*, 1999), has been shown to promote exosome release from human leukemia K562 cells (Fader *et al.*, 2009), VAMP7 inhibition has been reported not to affect exosome release from epithelial Madin-Darby canine kidney (MDCK) cells (Proux-Gillardeaux *et al.*, 2007). Thus, no general SNARE proteins, paticularly specific SNARE complexes, that directly mediate the MVB–plasma membrane fusion have been identified.

In this study, we systematically screened for SNARE proteins that mediate exosome release and succeeded in identifying the R-SNARE VAMP5 as an MVB-localized regulator of exosome release. Depletion of VAMP5 impaired exosome release from HeLa cells. We also found that VAMP5 forms two distinct SNARE complexes with SNAP47 and STX1 or STX4 to mediate exosome release. In addition, we showed that VAMP5 and distinct sets of Q-SNAREs differently mediate apical and basolateral exosome release from polarized MDCK cells. Our findings suggest that VAMP5 is a general central regulator of exosome release from various cell types.

## Materials and Methods

### Cell culture

HeLa, HEK293T, and MDCK-II cells were cultured in Dulbecco’s modified Eagle’s medium (DMEM) (Fujifilm Wako Pure Chemical, Osaka, Japan) supplemented with 10% fetal bovine serum (FBS), 100 U/ml penicillin and 100 μg/ml streptomycin in a 5% CO_2_ incubator. To purify sEVs or exosomes released from polarized MDCK cells, 1 × 10^6^ MDCK cells were plated on cell culture inserts (140640, Thermo Fisher Scientific, Waltham, MA, USA). The culture medium was changed every 24 h, and after 3 days the cells were washed twice with PBS and once with DMEM without FBS. The cells were then cultured for 24 h in 1 ml of DMEM containing 1% EV-depleted FBS. EV-depleted FBS was obtained by ultracentrifugation at 100,000 × *g* for 24 h and filtration through a 0.22-μm filter.

### Plasmids

pMRX-IRES-blast-human CD63 was prepared as described previously (Matsui *et al.*, 2021). Mouse or human SNARE cDNAs were amplified by PCR using mouse or human brain Marathon-Ready cDNA as a template (Clontech/Takara Bio, Shiga, Japan). The cDNAs were inserted into pMRX-IRES-puro together with enhanced GFP (EGFP), 3 × FLAG, or 3 × Myc. SNARE expression plasmids are available from RIKEN BioResource Research Center in Japan (https://dnaconda.riken.jp/search/depositor/dep005893.html; Cat# RDB20035–RDB20073).

### Antibodies and reagents

All primary antibodies used in this study are listed in Table S1. Rabbit polyclonal anti-podocalyxin antibody was prepared as described previously (Mrozowska and Fukuda, 2016). Horseradish peroxidase (HRP)-conjugated anti-mouse IgG goat polyclonal antibody (Southern Biotech, Birmingham, AL, USA), HRP-conjugated anti-rabbit IgG donkey antibody (GE Healthcare, Chicago, IL, USA), HRP-conjugated Protein-G (Abcam, Cambridge, UK), and Alexa Fluor 488/555/633-conjugated anti-goat/mouse/rat IgG donkey or goat polyclonal antibodies (Thermo Fisher Scientific) were used as secondary antibodies. Alexa Fluor 488-conjugated Phalloidin (Thermo Fisher Scientific) was used to visualize actin.

### Isolation of CD63-positive sEVs (mainly exosomes) from cell culture medium

The media collected were first subjected to a centrifugation step at 700 × *g* for 5 min to pellet and remove cells, and the supernatant was spun at 3,000 × *g* for 10 min to remove cell debris and apoptotic bodies. The second supernatant was centrifuged at 16,500 × *g* for 30 min to remove heavy microvesicles, and any remaining large EVs were removed by passing the final supernatant through a 0.22-μm pore PES filter (Millipore, Burlington, MA, USA). The supernatant (pre-cleared medium) obtained was then subjected to direct immunoaffinity capture to remove Annexin I-positive small vesicles (i.e., microvesicles). Direct immunoaffinity capture was performed as described previously (Jeppesen *et al.*, 2019). In brief, the pre-cleared medium was incubated, with rotation, for 16 h at 4°C with Dynabeads (Thermo Fisher Scientific) directly conjugated to anti-CD63 antibody. The beads were then washed twice with 0.22-μm filtered ice-cold 0.1% BSA-PBS and washed once with 0.22-μm filtered PBS. Immediately following the final wash, the exosome-loaded beads were suspended in an SDS sample buffer without reducing agent (referred to as the exosome sample throughout this study, unless otherwise specified). The beads were removed from the suspension with a magnet, and the clarified lysates were used for immunoblotting. For the NTA, exosomes were eluted from the beads by incubating the exosome-loaded beads in 0.22-μm filtered 0.1 M glycine-HCl buffer (pH 3.0) for 5 min at room temperature and then equilibrating with a one-fourth volume of 0.22-μm filtered 1 M Tris-HCl (pH 8.0). The supernatant that remained after removing the beads with the magnet was suspended in 0.22-μm filtered PBS.

### Retroviral infections and generation of stable cell lines

HEK293T cells were transiently transfected with retrovirus vectors, pCG-gag-pol, and pCG-VSV-G (provided by T. Yasui, The National Institute of Biomedical Innovation, Health and Nutrition, Osaka, Japan) by using Lipofectamine 2000 (Thermo Fisher Scientific). Two days after transfection, culture medium containing retrovirus was collected and filtered through a 0.22-μm pore PES filter. HeLa cells and MDCK cells were cultured with retrovirus and 8 μg/ml polybrene, and uninfected cells were removed with 5 μg/ml blasticidin S and/or 1 μg/ml puromycin.

### Establishment of *VAMP5*-KO HeLa cells

CRISPR guide RNA (gRNA) sequences designed to target the human *VAMP5* gene were cloned into the pSpCas9(BB)-2A-bsr vector (Oguchi *et al.*, 2020). The target sequence was human *VAMP5*, 5'-GCTCCAACTCTATTCCTGCC-3'. HeLa cells were transfected using Lipofectamine 2000 and the pSpCas9(BB)-2A-bsr vector carrying the above gRNA sequence. After 24 h, untransfected cells were removed with 5 μg/ml blasticidin S. Clones with mutations in both alleles were identified by genomic DNA sequencing and immunoblotting. Two independent *VAMP5*-KO cell lines, both of which showed decreased exosome secretion, were obtained, and #13 clone was used in this study. A single-nucleotide insertion (boldface; 5'-GGC**A**AGGAATAGAGTTGGAGC-3') or a single-nucleotide deletion and a single-nucleotide substitution (hyphen and underline, respectively; 5'-GG-AGGTATAGAGTTGGAGC-3') was found in the target sequence of the #13 clone.

### RNAi

siRNA oligonucleotides were purchased from Nippon Gene (Tokyo, Japan) and Thermo Fisher Scientific. The target sequences used are listed in Table S1. Cells were transfected with the siRNA oligonucleotides by using Lipofectamine RNAiMAX (Thermo Fisher Scientific) according to the manufacturer’s instructions.

### Immunoprecipitation and immunoblotting

Cell lysates were prepared in a lysis buffer (50 mM HEPES-KOH, pH 7.2, 150 mM NaCl, 1% Triton X-100, and protease inhibitor cocktail [cOmplete^TM^, EDTA-free protease inhibitor cocktail; Roche, Basel, Switzerland]). After centrifugation at 16,500 × *g* for 10 min, the supernatants were subjected to immunoprecipitation by using anti-FLAG M2 affinity gel (Sigma-Aldrich, St. Louis, MO, USA). The immunocomplexes obtained were washed three times in a washing buffer (50 mM HEPES-KOH, pH 7.2, 150 mM NaCl, and 1% Triton X-100) and boiled in an SDS sample buffer. Samples were subsequently separated by SDS-PAGE and transferred to Immobilon-P polyvinylidene difluoride membranes (Millipore). Immunoblot analyses were performed with the antibodies indicated, and visualization was achieved with the Immobilon Western Chemiluminescent HRP substrate (Millipore).

### Isolation of CD63-positive MVBs from HeLa cells

HeLa cells stably expressing CD63-3 × FLAG were suspended with a homogenization buffer (20 mM HEPES-KOH, pH 7.2, 40 mM sucrose, 1 mM EDTA and protease inhibitor cocktail) and homogenized through a 25-gauge needle. After centrifugation at 3,000 × *g* for 10 min, the supernatants were subjected to immunoprecipitation for 4 h by using anti-DYDDDDK-tag antibody magnetic beads (Fujifilm Wako Pure Chemical). The CD63-3 × FLAG-positive MVBs obtained were washed three times in a homogenization buffer, and immediately after the final wash, the MVB-loaded beads were suspended in an SDS sample buffer without reducing agent. The beads were removed from the suspension with a magnet, and the clarified lysates were used for immunoblotting.

### Nanoparticle tracking analysis (NTA)

Purified exosome samples were analyzed for particle concentration and size distribution by using the NTA method described by Malvern NanoSight NS300 (Malvern Panalytical, Malvern, UK). The assays were performed according to the protocol recommended by the manufacturer. Briefly, three independent replicates of exosome preparations diluted in 0.22-μm filtered PBS were injected at a constant rate into the tracking chamber with the syringe pump provided, and the specimens were tracked at room temperature for 60 s. Shutter and gain were manually adjusted for optimal detection and maintained at the optimized settings for all samples. The data were captured and analyzed with NTA software (version 3.4, Malvern Panalytical). Under our experimental conditions, the majority of the CD63-positive exosomes were recovered in 50–100 nm size fractions (i.e., typical exosome size), but the peak value often shifted to a lower or higher value and split peaks were sometimes observed in each trial, even when the control sample was used. Because of this technical issue, we did not consider the peak shifts or splits within the 50–100 nm size range in this study to be significant. In addition to exosomes of typical size, we often observed larger vesicles measuring 100–150 nm, and their numbers differed from experiment to experiment, even in the control samples. These larger vesicles may be aggregates of more than two exosomes that were formed artificially during the purification procedure.

### Immunocytochemistry

Cells grown on coverslips (Kishiwada, Osaka, Japan) or glass-bottom dishes (MatTek, Ashland, MA, USA) were washed with PBS and fixed in 4% PFA or 10% TCA for 10 min at room temperature. The fixed cells were permeabilized with 50 μg/ml digitonin (Sigma-Aldrich) in PBS for 5 min, blocked with 3% BSA in PBS for 30 min, and then incubated with primary antibodies for 1 h. After washing three times with PBS, the cells were incubated with Alexa Fluor 488/555/633-conjugated anti-donkey or goat IgG secondary antibodies for 1 h. The coverslips were examined with FV1000 IX81 (Olympus, Tokyo, Japan) equipped with a 100× oil-immersion objective lens (1.45 NA, Olympus) and BZ-X810 (Keyence, Osaka, Japan) equipped with a 60× oil-immersion objective lens (1.40 NA, Nikon, Tokyo, Japan). The images were processed by using Photoshop 2022 software (Adobe, San Jose, CA, USA).

### EGF receptor (EGFR) degradation assay

HeLa cells were cultured at 90% confluence in serum free DMEM for 20 h and then incubated with DMEM containing 200 ng/ml EGF (Thermo Fisher Scientific) for the times shown in Fig. S2C. Cell lysates were analyzed by immunoblotting with the antibodies indicated.

### Electron microscopy

Cells were cultured on cell tight C-2 cell disks (Sumitomo Bakelite, Tokyo, Japan) and fixed for 2 h in 2.5% glutaraldehyde (Electron Microscopy Sciences, Hartfield, PA, USA) in 0.1 M phosphate buffer, pH 7.4 on ice. The cells were washed with 0.1 M phosphate buffer, pH 7.4 three times, postfixed in 1% osmium tetroxide in 0.1 M phosphate buffer, pH 7.4 for 2 h, dehydrated, and embedded in Epon 812 according to the standard procedure. Ultrathin sections were stained with uranyl acetate and lead citrate. All samples were examined with a JEM-1400Flash electron microscope (JEOL, Akishima, Japan).

### Statistical analysis

Two groups of data were evaluated by the unpaired two-tailed Student’s *t*-test, and multiple comparisons were performed by one-way analysis of variance (ANOVA) followed by the Tukey’s test. The statistical analysis was performed with Prism9 (GraphPad software).

## Results and Discussion

### VAMP5 is an MVB-localized R-SNARE

**Fig. 1:**
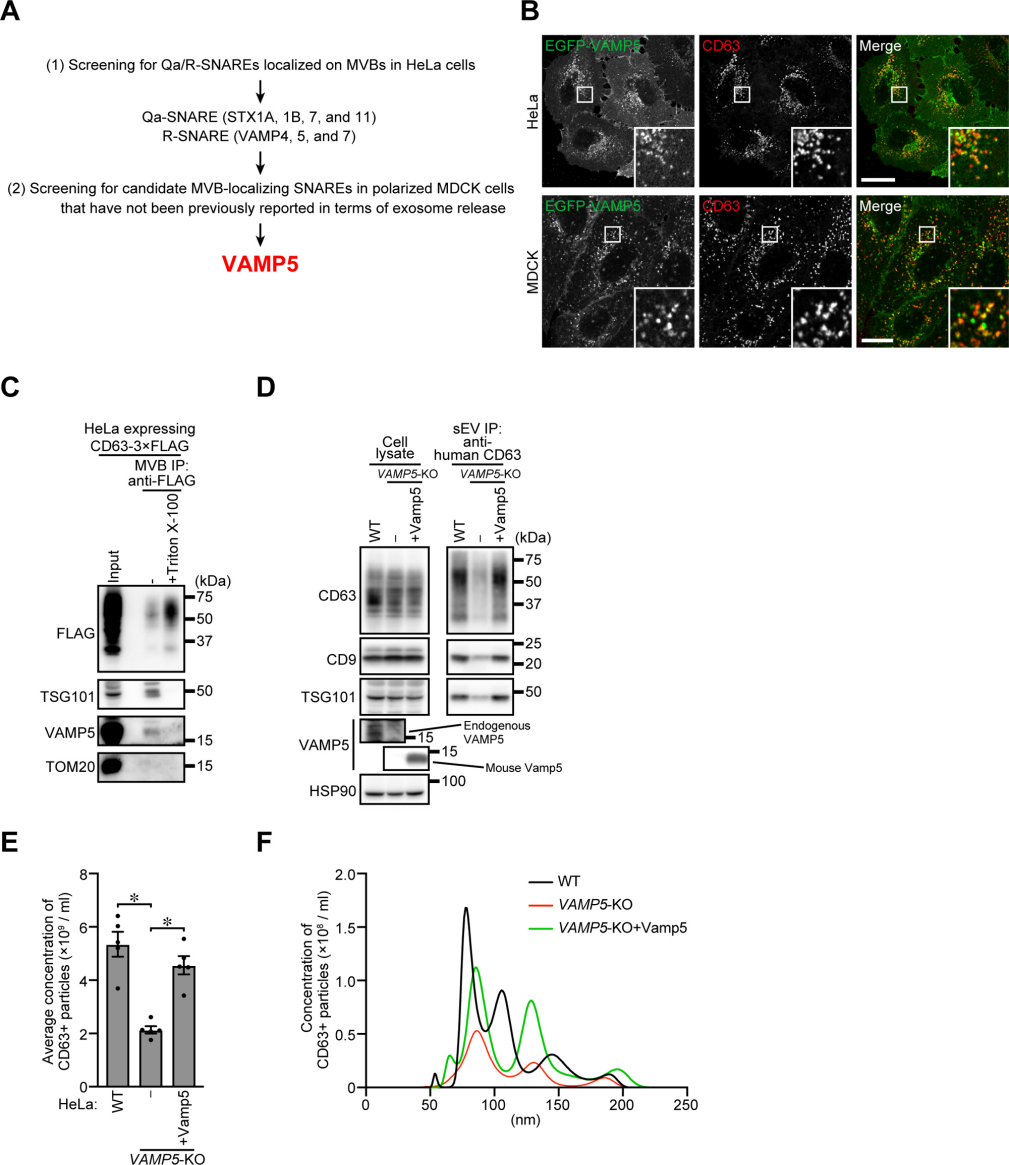
MVB-resident VAMP5 is required for exosome release from HeLa cells (A) Strategy to identify Qa/R-SNARE proteins that are present at MVBs. (B) HeLa and MDCK cells stably expressing EGFP-VAMP5 were immunostained with anti-CD63 antibody. Scale bars, 20 μm. (C) HeLa cells stably expressing CD63-3 × FLAG were homogenized without detergent, and CD63-positive MVBs were immuno-isolated from the homogenate with anti-FLAG antibody. Total homogenate and MVB proteins were analyzed by immunoblotting with the antibodies indicated. It was noteworthy that VAMP5 was detected in the MVB sample only in the absence of Triton X-100, suggesting that endogenous VAMP5 localizes at MVBs. (D) WT, *VAMP5*-KO, and rescued HeLa cells cultured for 48 h in the completed medium. Then, the culture medium was replaced with EV-depleted medium. One day later, exosomes were isolated by direct immunoaffinity capture with anti-CD63 antibody. Cell lysates and the exosomal proteins isolated were analyzed by immunoblotting with the antibodies indicated. (E) Exosomes prepared as in (D) were eluted from the beads with a glycine buffer and analyzed by NTA. Quantification of the NTA data obtained in five independent experiments. *P<0.01 (one-way ANOVA and Tukey’s test). Means ± s.e.m. are shown. (F) Representative NTA traces are shown.

To identify SNAREs directly involved in exosome release, we first screened for Qa- or R-SNAREs that are present at MVBs, because when a Qa-SNARE is present at MVBs, its partner R-SNARE should be on the plasma membrane and vice versa. To this end, we established HeLa cells stably expressing each EGFP-tagged mammalian Qa- or R-SNARE and investigated its colocalization with the MVB marker CD63. Of the 25 Qa- and R-SNAREs investigated, 7 SNAREs (STX1A, STX1B, STX11, VAMP4, VAMP5, VAMP7, and SEC22C) were well colocalized with CD63 in HeLa cells (Fig. 1A; and Fig. S1A and S1B). Because, as mentioned above in the introduction, VAMP7 appears to be involved in exosome release from only a specific cell line (Fader *et al.*, 2009; Proux-Gillardeaux *et al.*, 2007) and our preliminay data showed that depletion of VAMP7 in HeLa cells rather increased exosome release (data not shown), and thereby we excluded VAMP7 from the candidates. Of the remaining 6 candidate SNAREs, we found that VAMP5 is most extensively co-localized with CD63 in polarized MDCK cells (Fig. 1B; and Fig. S1C and S1D). Moreover, endogenous VAMP5 was also detected in the isolated CD63-positive MVB sample (Fig. 1C). Thus, VAMP5 is likely to be an MVB-localized SNARE protein. VAMP5 is a ubiquitously expressed SNARE protein and contains no specific domains except its SNARE domain and transmembrane region (Zeng *et al.*, 1998). VAMP5 has been shown to be required for glucose transporter 4 (GLUT4) translocation to the plasma membrane (Schwenk *et al.*, 2010), and *Vamp5*-KO mice have a low birth rate and exhibit urinary and respiratory systems abnormalities (Ikezawa *et al.*, 2018). However, since nothing was known about the role of VAMP5 in exosome release, we selected it as a prime candidate for a novel MVB-localized SNARE that generally regulates exosome release and investigated the impact of its depletion on exosome release.

### VAMP5 is required for exosome release from HeLa cells

To determine whether VAMP5 is involved in exosome release, we established *VAMP5*-KO HeLa cells by using the CRISPR/Cas9 system and then collected CD63-positive exosomes from the culture medium of wild-type (WT) cells, *VAMP5*-KO cells, and rescued cells (*VAMP5*-KO cells stably expressing mouse Vamp5) by direct immunoaffinity capture with anti-CD63 antibody (Jeppesen *et al.*, 2019). As shown in Fig. 1D, *VAMP5*-KO dramatically decreased the amounts of CD63, CD9, and TSG101 in the exosome samples, and this phenotype was rescued by re-expression of mouse Vamp5. The results of a nanoparticle tracking analysis (NTA) also showed that exosome release from *VAMP5*-KO HeLa cells was significantly reduced with little or no effect on exosome size (mainly around 50–100 nm) (Fig. 1E and 1F).

To exclude the possibility that *VAMP5*-KO affects exosome biogenesis, and/or MVB formation and function rather than exosome release itself, we performed an immunofluorescence and an electron microscopic analysis of MVBs. As expected, no change in MVB localization was observed in *VAMP5*-KO HeLa cells compared with WT cells (Fig. S2A). The electron microscopic analysis also confirmed normal MVB morphology and size in *VAMP5*-KO HeLa cells (Fig. S2B), suggesting that MVB formation occurred normally in the KO cells. In addition, an EGFR degradation assay showed that the endocytic pathway was unaffected by *VAMP5*-KO (Fig. S2C). Taken together, these findings indicated that VAMP5 regulates exosome release without affecting exosome biogenesis.

### SNAP47, a VAMP5-binding Qbc-SNARE protein, is required for exosome release

**Fig. 2:**
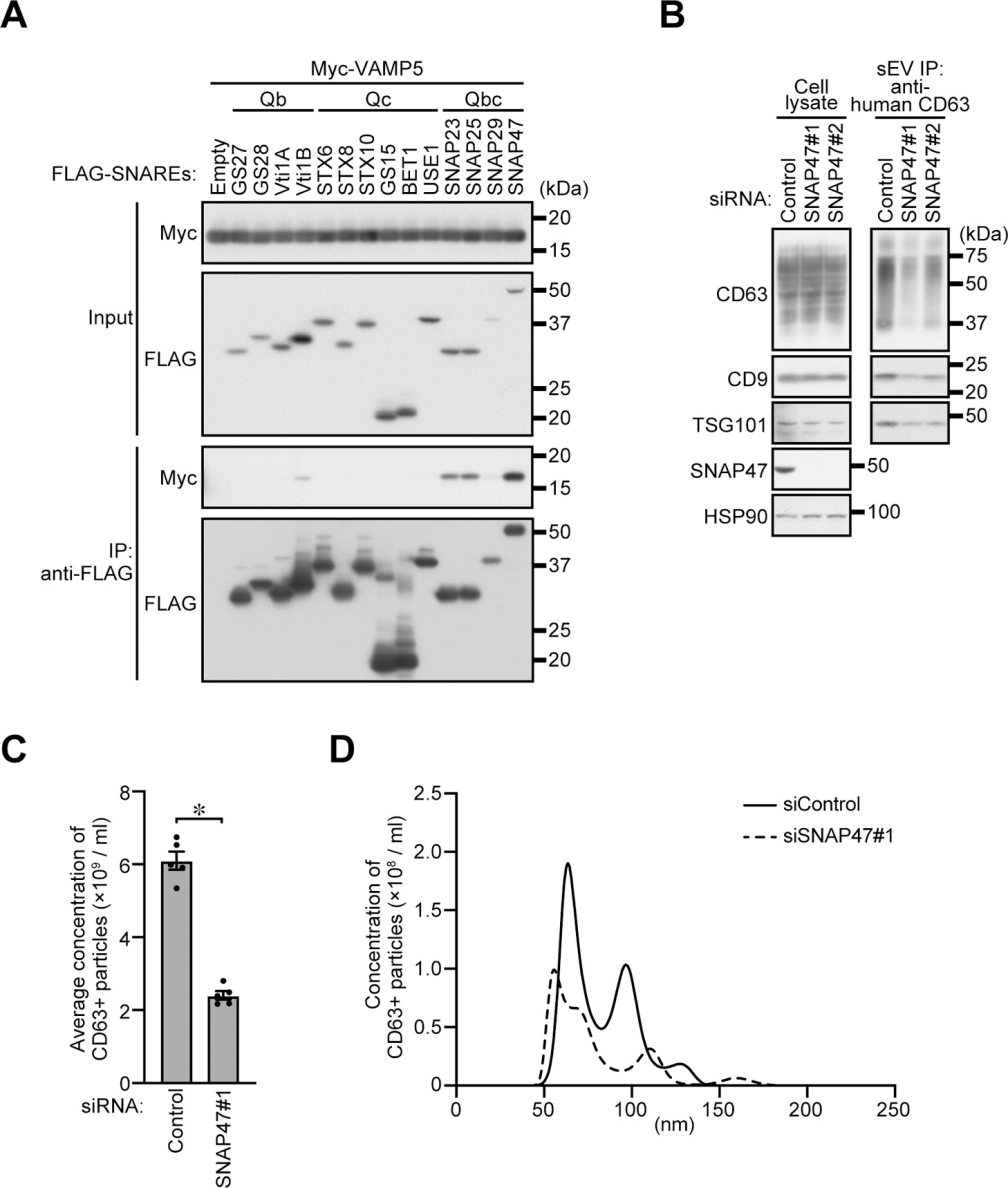
SNAP47, a VAMP5-binding Qbc-SNARE, mediates exosome release from HeLa cells (A) HEK293T cells transiently expressing 3 × FLAG-tagged Qb-, Qc-, or Qbc-SNAREs and/or 3 × Myc-VAMP5 were lysed, and the cell lysates were immunoprecipitated with anti-FLAG antibody. (B) HeLa cells were transfected with siControl or siSNAP47s. After 48 h, the culture medium was replaced with EV-depleted medium. One day later, exosomes were isolated by direct immunoaffinity capture with anti-CD63 antibody. Cell lysates and the exosomal proteins isolated were analyzed by immunoblotting with the antibodies indicated. (C) Exosomes prepared as in (B) were eluted from the beads with a glycine buffer and analyzed by NTA. Quantification of the NTA data obtained in five independent experiments. *P<0.01 (unpaired two-tailed Student’s *t*-test). Means ± s.e.m. are shown. (D) Representative NTA traces are shown.

Since VAMP5 is an R-SNARE, it should interact with Qa-, Qb-, and Qc-SNAREs to mediate SNARE-catalyzed membrane fusion (Hong, 2005; Jahn and Scheller, 2006). Among the Qb- and Qc-SNAREs tested, VAMP5 was able to interact with Vti1B (Qb-SNARE), SNAP23, SNAP25, and SNAP47 (Qbc-SNAREs; Fig. 2A), but since SNAP47 most strongly interacted with VAMP5, we focused on SNAP47 among them. Although SNAP47 had been reported to be present at CD63-positive endosomes (Kuster *et al.*, 2015) and to be involved in neuronal secretion (Urbina *et al.*, 2021) and autophagy (Aoyagi *et al.*, 2018; Corona *et al.*, 2018), its involvement in exosome release had never been investigated.

We therefore proceeded to examine the effect of SNAP47 knockdown on exosome release from HeLa cells. As shown in Fig. 2B, SNAP47 knockdown reduced the amounts of CD63, CD9, and TSG101 in the exosome samples, the same as the *VAMP5*-KO described above (Fig. 1D). Similarly, depletion of SNAP47 significantly decreased the number of exosomes (Fig. 2C and 2D). Moreover, the subcellular localization and size of the MVBs were unaffected in SNAP47-knockdown HeLa cells (Fig. S3A). These findings indicated that SNAP47 regulates exosome release presumably together with VAMP5.

### Identification of SNAP47-binding and plasma membrane-localized Qa-SNAREs

**Fig. 3:**
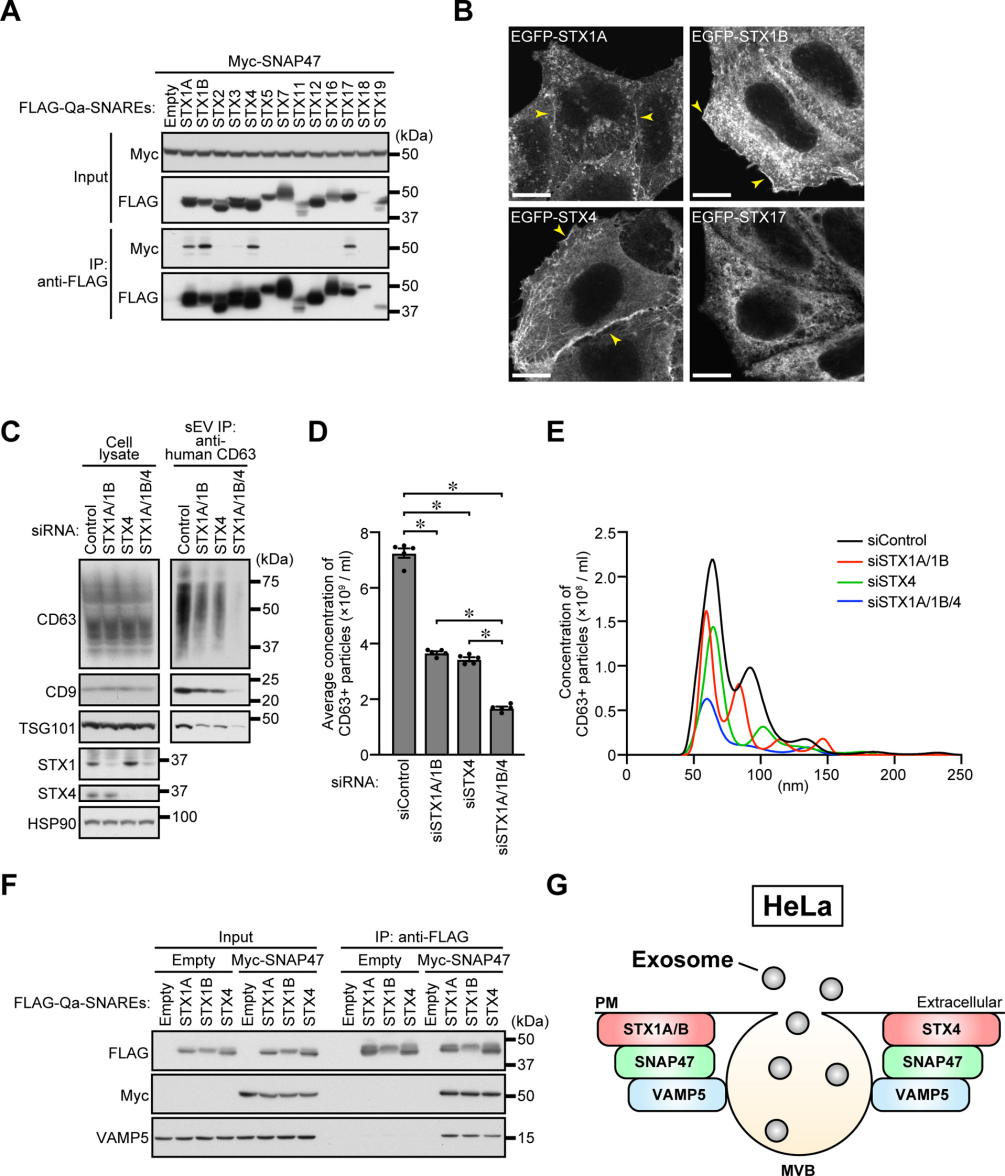
SNAP47-binding Qa-SNARE STX1A/1B/4 are required for exosome release from HeLa cells (A) HEK293T cells transiently expressing 3 × FLAG-Qa-SNAREs and/or 3 × Myc-SNAP47 were lysed, and the cell lysates were immunoprecipitated with anti-FLAG antibody. (B) HeLa cells stably expressing EGFP-tagged STX1A, STX1B, STX4, or STX17 were fixed and examined by confocal microscopy. The plasma membrane localization of each SNARE is indicated by yellow arrowheads. Scale bars, 20 μm. (C) HeLa cells were transfected with siControl or the siRNAs indicated. After 48 h, the culture medium was replaced with EV-depleted medium. One day later, exosomes were isolated by direct immunoaffinity capture with anti-CD63 antibody. Cell lysates and the exosomal proteins isolated were analyzed by immunoblotting with the antibodies indicated. (D) Exosomes prepared as in (C) were eluted from the beads with a glycine buffer and analyzed by NTA. Quantification of the NTA data obtained in five independent experiments. *P<0.01 (one-way ANOVA and Tukey’s test). Means ± s.e.m. are shown. (E) Representative NTA traces are shown. (F) HEK293T cells transiently expressing 3 × FLAG-STX1A/1B/4 and/or 3 × Myc-SNAP47 were lysed, and the cell lysates were immunoprecipitated with anti-FLAG antibody. (G) Proposed model of VAMP5-mediated exosome release from HeLa cells.

We next sought to identify a Qa-SNARE that functions together with VAMP5 and SNAP47 by performing binding assays with mammalian Qa-SNAREs, and the results showed that SNAP47 interacted with STX1A, STX1B, STX4, and STX17 (Fig. 3A). Since VAMP5 was predominantly localized at MVBs (Fig. 1B), we searched for Qa-SNAREs that localized to the plasma membrane and identified STX1A, STX1B, and STX4 as candidates (Fig. 3B). STX1A/B (hereafter referred to as STX1) and STX4 are well-known plasma membrane SNAREs (Jahn and Scheller, 2006; Teng *et al.*, 2001), and they are involved in various types of exocytosis, including neurotransmitter release (STX1; Bennett *et al.*, 1992), insulin secretion (STX1; Martin *et al.*, 1995), and translocation of GLUT4 and glutamate receptors to the plasma membrane (STX4; Bin *et al.*, 2018; Tamori *et al.*, 1998). STX1A and STX4 have recently been shown to be involved in exosome release in *Drosophila* cells and human cells, respectively (Gonzalez-Mendez *et al.*, 2020; Koles *et al.*, 2012; Verweij *et al.*, 2018), and under our experimental conditions STX1 or STX4 knockdown in HeLa cells significantly inhibited exosome release (Fig. 3C–3E) without affecting MVB morphology or distribution (Fig. S3A). Depletion of both STX1 and STX4 showed an additive effect on exosome release (Fig. 3C–3E), strongly suggesting that STX1 and STX4 mediate exosome release independently of each other.

We next investigated whether VAMP5 forms a complex with SNAP47 and STX1A, STX1B, or STX4. The results of our immunoprecipitation assays showed that endogenous VAMP5 co-precipitated with FLAG-STX1A/1B/4 together with Myc-SNAP47 only under Myc-SNAP47-expressing conditions (Fig. 3F), suggesting that VAMP5 can form a ternary complex with SNAP47 and STX1A/1B/4. These findings allowed us to conclude that the MVB-resident R-SNARE VAMP5 regulates exosome release from HeLa cells together with Qbc-SNARE SNAP47 and plasma membrane Qa-SNARE STX1 or STX4 (Fig. 3G).

### VAMP5 is required for polarized exosome release from epithelial MDCK cells

To evaluate the importance of the existence of two distinct sets of Q-SNAREs that regulate exosome release from single cells, we focused our attention on a polarized epithelial cell line MDCK, because we previously showed that polarized MDCK cells asymmetrically releases two distinct types of exosomes from the apical and basolateral membrane (named apical exosomes and basolateral exosomes, respectively) (Matsui *et al.*, 2021). These exosomes have different protein compositions and are originated from different MVBs. We also showed that each secretory MVBs are transported to an apical membrane or basolateral membrane by a distinct mechanism (Matsui *et al.*, 2022). However, SNARE complexes that mediate the MVB–apical or MVB–basolateral membrane fusion have not yet been identified.

**Fig. 4:**
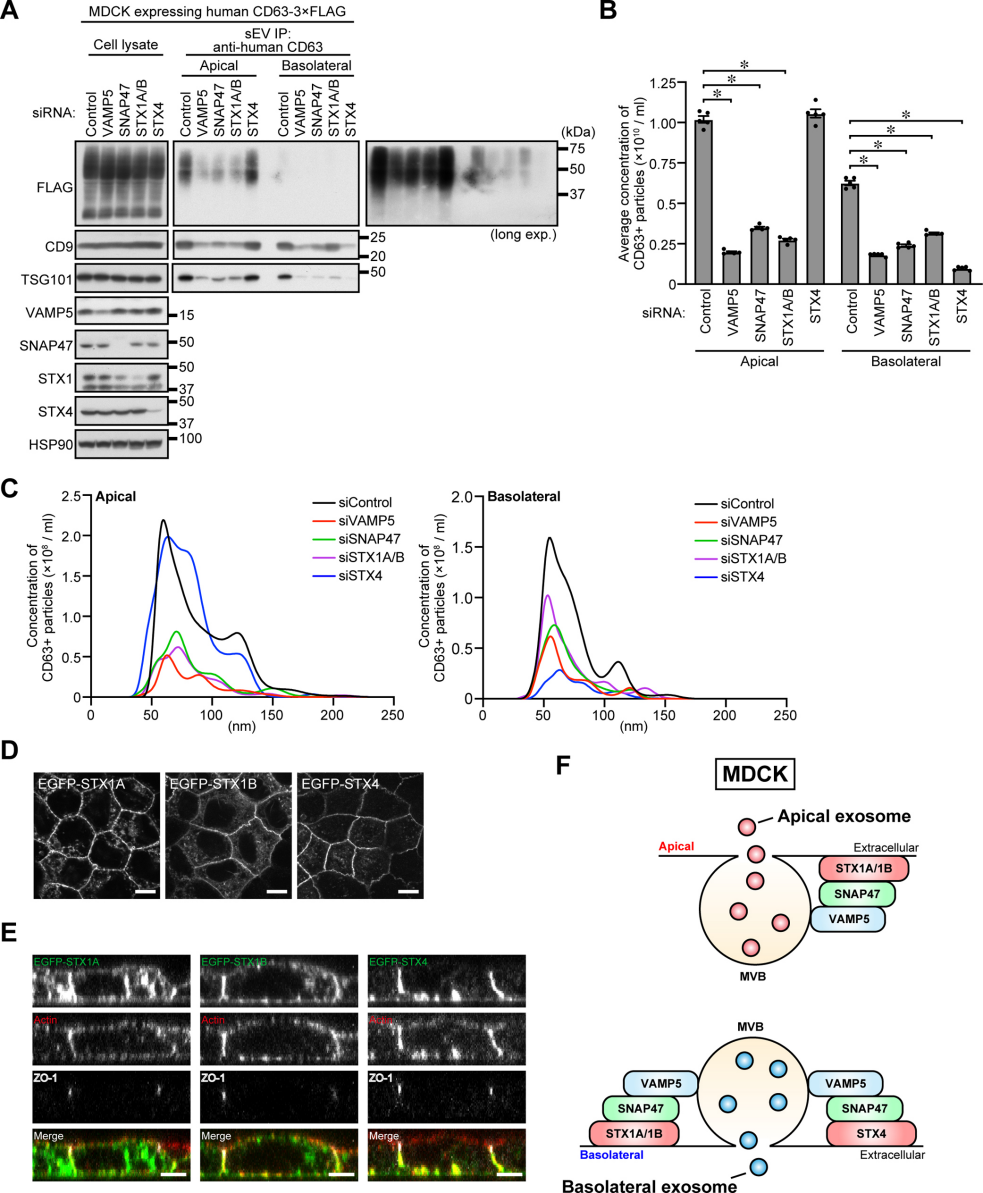
VAMP5, SNAP47, or STX1A/1B/4 are required for exosome release from polarized MDCK cells (A) MDCK cells were transfected with siControl or the siRNAs indicated, and the cells were transferred to cell culture inserts and cultured for 4 days. On the last day, the culture medium was replaced with EV-depleted medium. sEVs were isolated from the pre-cleared medium by direct immunoaffinity capture using anti-CD63 antibody. Cell lysates and the exosomal proteins isolated were analyzed by immunoblotting with the antibodies indicated. (B) Exosomes prepared as in (A) were eluted from the beads with a glycine buffer and analyzed by NTA. Quantification of the NTA data obtained in five independent experiments. *P<0.01 (one-way ANOVA and Tukey’s test). Means ± s.e.m. are shown. (C) Representative NTA traces are shown. (D) MDCK cells stably expressing EGFP-STX1A/1B/4 were cultured for 72 h. The cells were then fixed and examined by confocal microscopy. Scale bars, 20 μm. (E) The cells were cultured as in (D) and immunostained with anti-ZO-1 antibody. Actin was visualized with phalloidin. Scale bars, 5 μm. (F) Proposed model of VAMP5-mediated exosome release from polarized MDCK cells.

In the final sets of experiments, we investigated whether VAMP5, SNAP47, and STX1 or STX4 are involved in the asymmetric exosome release from polarized MDCK cells. To do so, we established MDCK cells stably expressing human CD63 and separately collected apical and basolateral CD63-positive exosomes by direct immunoaffinity capture with anti-CD63 antibody. As shown in Fig. 4A, knockdown of either VAMP5, SNAP47, or STX1 in polarized MDCK cells decreased the amount of CD63, CD9, and TSG101 in both apical and basolateral exosome samples. STX4 knockdown, on the other hand, specifically reduced the amount of the basolateral exosomal proteins without altering the amount of protein in the apical samples. NTA yielded similar results (Fig. 4B and 4C). Importantly, depletion of either VAMP5, SNAP47, STX1, or STX4 itself had no effect on either MVB formation or localization (Fig. S3B). In addition, normal apical–basolateral asymmetry was established in each of the knockdown cells, the same as in the control cells: Podocalyxin (an apical membrane marker) signals were detected in the upper side (apical portion) of the cells, and ZO-1-positive tight junctions were also observed (Fig. S3C). These findings indicated that VAMP5, SNAP47, STX1, and STX4 mediate exosome release across different cell types, i.e., non-polarized human HeLa cells and polarized canine MDCK cells.

STX4 has been reported to be present only at the basolateral membrane of polarized MDCK cells (Low *et al.*, 1996). Consistent with this report, we confirmed that EGFP-STX4 was localized at the basolateral membrane, whereas EGFP-STX1A and -STX1B were localized at both sides of the plasma membrane (Fig. 4D and 4E). These findings taken together suggested that the VAMP5–SNAP47–STX1 complex mediates both apical and basolateral exosome release while the VAMP5–SNAP47–STX4 complex mediates only basolateral exosome release (Fig. 4F).

In this study, we systematically screened for SNARE proteins that are involved in exosome release, and we succeeded in identifing R-SNARE VAMP5 as a novel exosome regulator. We demonstrated that VAMP5 is present at MVBs and regulates exosome release through SNARE complex formation with SNAP47 and STX1 or STX4. Since STX1 and STX4 are likely to function independently of each other in HeLa cells (Fig. 3C–3E) and STX4 functions at only the basolateral side of MDCK cells (Fig. 4A–4C), we propose that two distinct SNARE complexes, i.e., VAMP5–SNAP47–STX1 and VAMP5–SNAP47–STX4, mediate MVB–plasma membrane fusion in human cells and canine cells (Fig. 3G and Fig. 4F)

Upon MVB–plasma membrane fusion, how are STX1 and STX4 differentially used? Exosomal heterogeneity, i.e., the release of various types of exosomes from a single cell, has recently been reported, but very little is known about the mechanisms by which they are released (Colombo *et al.*, 2013; Kalluri and LeBleu, 2020; Kowal *et al.*, 2016; Matsui *et al.*, 2021; Zhang *et al.*, 2018). Thus, it is possible that STX1 and STX4 are used for distinct types of VAMP5-positive MVBs that contain seeds of different exosomes (e.g., apical and basolateral exosomes) and thereby release heterogeneous exosomes.

A previous study indicated that STX5 is required for MVB–plasma membrane fusion in *C. elegans* cells and that its absence results in MVB accumulation under the plasma membrane (Hyenne *et al.*, 2015). However, no such phenotype was observed in SNAP47-, STX1A/B-, STX4-knockdown cells, or even in *VAMP5*-KO cells (Fig. S2A; and Fig. S3A and S3B). Furthermore, none of the exosomal proteins tested were observed to have accumulated in the cell lysates of any of the SNARE-depleted cells (e.g., *VAMP5*-KO HeLa cells in Fig. 1D), indicating that secretory MVBs are only a minor population of all MVBs in mammalian cells (less than 1% of all MVBs in the case of MDCK cells; Matsui *et al.*, 2021). Thus, it is not surprising that no MVB accumulation near the plasma membrane was observed in the mammalian cells even in the absence of VAMP5 or its cognate SNAREs.

When and how does VAMP5 recognizes and is recruited to secretory MVBs? A recent study has revealed that VAMP5 is present in the luminal side of MVBs and that VAMP5 itself is released as a component of CD63-positive sEVs or exosomes from Müller cells, a type of radial glial cells in the retina (Demais *et al.*, 2022). These findings suggest that VAMP5 can be present on the surface of MVBs before the formation of ILVs, the seeds of exosomes, and some of the MVB-localized VAMP5 may be accidentally incorporated into ILVs together with exosomal proteins such as CD63, thereby resulting in its secretion as exosomes. Similar inclusion of exosome regulators, e.g., ALIX, in exosomes has also been reported (Baietti *et al.*, 2012). Although the mechanism responsible for VAMP5 targeting to the MVB surface is unknown, we hypothesize that VAMP5 is translocated to the MVB surface via the endocytic pathway, because VAMP5 is partially localized at the plasma membrane (Fig. 1B; and Zeng *et al.*, 1998). Consistent with our hypothesis, late endosomal/lysosomal VAMP7 is known to be targeted to MVBs from the plasma membrane through the endocytic pathway, which is regulated by the specific interaction of an N-terminal longin domain of VAMP7 and Hrb (Pryor *et al.*, 2008). Thus, in the future it will be interesting to search for a VAMP5 interactor that is required for the MVB targeting, although, unlike VAMP7, VAMP5 consists of a SNARE domain and transmembrane region alone, and does not contain any additional domains (Zeng *et al.*, 1998).

Although exosome release is a universally conserved phenomenon from yeasts to human, VAMP5 is mostly conserved in vertebrates. Since exosome release was retained to some extent even in *VAMP5*-KO cells (Fig. 1D and 1E) and in VAMP5-knockdown cells (Fig. 4A–4C), VAMP5-mediated exosome release is not the sole mechanism, and there must also be an as yet unidentified VAMP5-independent mechanism. However, depletion of VAMP5 or its cognate Q-SNAREs resulted in a ~50%–80% reduction in exosome release from HeLa cells and MDKC cells. Thus, as far as we have been able to determine based on a search of the literature, VAMP5-mediated exosome release is the first and major mechanism of exosome release to have been identified in mammals. Further extensive studies will be necessary to identify additional VAMP5-independent exosome release mechanism(s).

## Author Contributions

T.M. and M.F. designed the experiments, interpreted the data, and wrote the manuscript. T.M. and S.H. carried out the experiments and interpreted the data. Y.S. performed EM analysis. All authors discussed the results and commented on the manuscript.

## Conflicts of Interest

The authors declare no competing interests.

## Abbreviations

DMEM: Dulbecco’s modified Eagle’s medium
EGFR: EGF receptor
EGFP: enhanced GFP
ER: endoplasmic reticulum
ILV: intraluminal vesicle
FBS: fetal bovine serum
GLUT4: glucose transporter 4
HRP: horseradish peroxidase
gRNA: guide RNA
MDCK: Madin-Darby canine kidney
MVB: multivesicular body
NTA: nanoparticle tracking analysis
sEV: small extracellular vesicle
SNARE: soluble *N*-ethylmaleimide-sensitive factor attachment protein receptor
STX: Syntaxin
WT: wild-type
