# Supplementary material for: VAMP5 and distinct sets of cognate Q-SNAREs mediate exosome release
Source: Cell Struct Funct. 2023 Sep 14;48(2):187–98. doi: 10.1247/csf.23067 (PMC11496780; doi:10.1247/csf.23067)
Supplement: Supplementary file 1 — Supplementary Materials [file csf_48_23067_1.pdf]

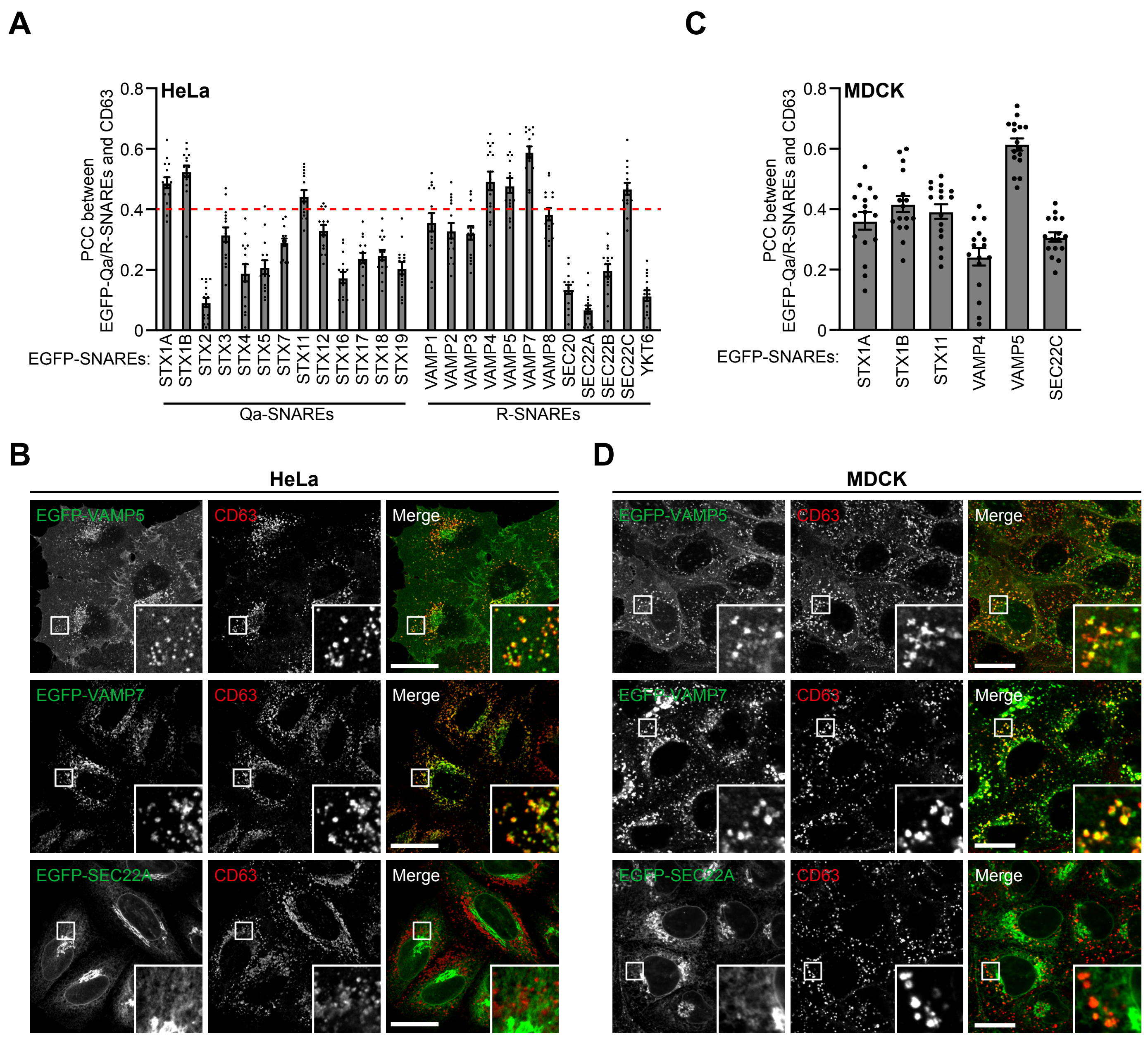

Figure S1. Identification of MVB-resident Qa/R-SNARE proteins.

(A) HeLa cells stably expressing EGFP-tagged Qa- or R-SNAREs were immunostained with anti-CD63 antibody. Quantifications (determined in 15 cells) of the ratios of colocalization of EGFP-SNAREs to CD63 are shown.

(B) Representative images of (A) are shown. Scale bars, 20 μm.

(C) Polarized MDCK cells stably expressing EGFP-candidate SNAREs were immunostained with anti-CD63 antibody. Quantifications (determined in 15 cells) of the ratios of colocalization of EGFP-SNAREs to CD63 are shown.

(D) Representative images of (C) are shown. Scale bars, 20 μm.

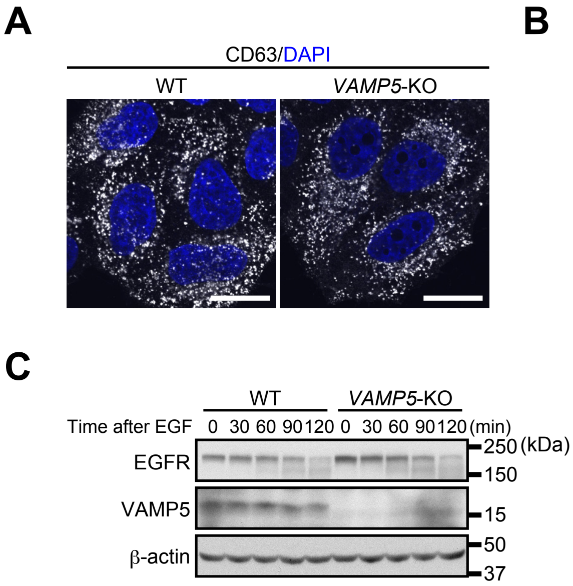

Figure S2. MVB function and morphology in *VAMP5*-KO HeLa cells are normal.

(A) WT and *VAMP5*-KO HeLa cells were immunostained with anti-CD63 antibody. Scale bars, 20 μm.

(B) WT and *VAMP5*-KO HeLa cells were examined by conventional electron microscopy. Scale bars, 200 nm.

(C) WT and *VAMP5*-KO HeLa cells were cultured in serum-free DMEM for 24 h and then treated with 200 ng/ml EGF for the times indicated. Cell lysates were analyzed by immunoblotting with the antibodies indicated.

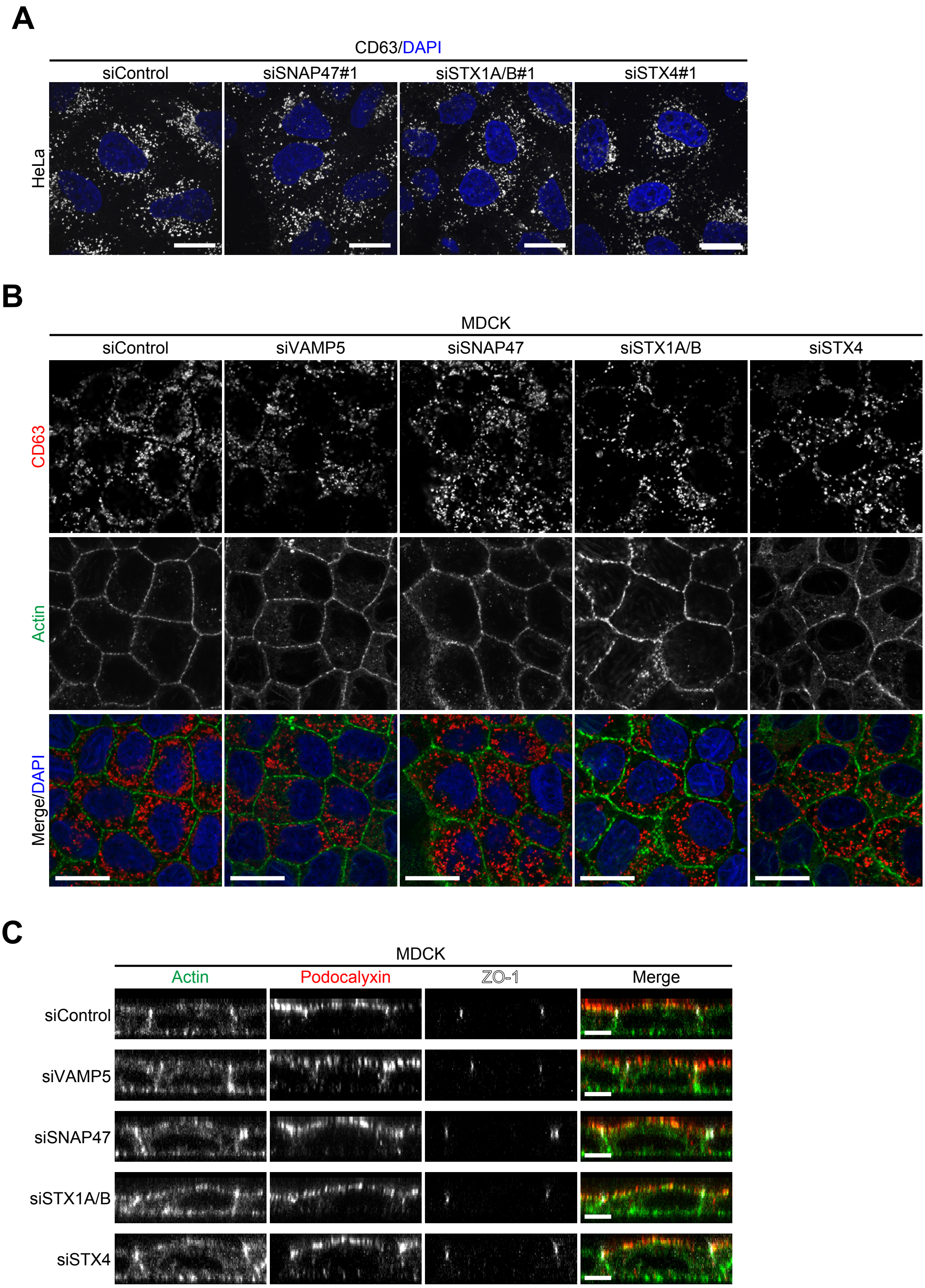

Figure S3. Effects of candidate SNARE-knockdown on MVB morphology and distribution, and polarization of MDCK cells.

(A) HeLa cells were transfected with siControl or the siRNAs indicated. After 48 h, the cells were immuno-stained with anti-CD63 antibody. Scale bars, 20 μm.

(B) MDCK cells were transfected with siControl or the siRNAs indicated. After 72 h, the cells were immuno-stained with anti-CD63 antibody. Actin was visualized with phalloidin. Scale bars, 20 μm.

(C) The cells were cultured as in (B) and immunostained with anti-podocalyxin and anti-ZO-1 antibodies. Actin was visualized with phalloidin. Scale bars, 5 μm.

Table S1. List of materials used in this study

Antibodies		
Target protein	Company	Product ID
CD63 for sEV IP	COSMO BIO	SHI-EXO-M02
CD63 for WB and IF (human)	Novus Biologicals	NBP2-42225
CD63 for IF (dog)	SICGEN	AB0047-200
CD9	Thermo Fisher Scientific	MA1-80307
TSG101	Santa Cruz Biotechnology	sc-136111
HSP90	BD	610419
β-actin (HRP-conjugated)	Proteintech	HRP-60008
VAMP5	MyBioSource	MBS9134376
SNAP47	ORIGENE	TA344342
STX1	Santa Cruz Biotechnology	sc-12736
STX4	Proteintech	14988-1-AP
GFP	Roche	11 814 460 001
FLAG (HRP-conjugated)	Sigma-Aldrich	A8592
Myc (HRP-conjugated)	MBL	M047-7
TOMM20	Santa Cruz Biotechnology	sc-136211
EGFR	Fitzgerald	20-ES04
Podocalyxin	Mrozowska and Fukuda, 2016	
ZO-1	Santa Cruz Biotechnology	sc-33725

siRNAs				
Name	Company	Product ID	Target gene	Target sequence
siControl	Nippon gene		Lusiferase	CGUACGCGGAAUACUUCGA
siSNAP47#1(human)	Thermo Fisher Scientific	SNAP47HSS133199	human SNAP47	CCCACAGAACAGAGUCUCACGUUAA
siSNAP47#2(human)	Thermo Fisher Scientific	SNAP47HSS133200	human SNAP47	GCAAGAAGAUGGAGCUGUUAGAAGA
siSTX1A#1(human)	Thermo Fisher Scientific	s13589	human STX1A	CAAACAAAGUUCGUUCCAA
siSTX1A#2(human)	Thermo Fisher Scientific	s13590	human STX1A	GAACUCAUGUCCGACAUAA
siSTX1B#1(human)	Thermo Fisher Scientific	s2223220	human STX1B	CAGAUGACAUCAAAAUGGA
siSTX1B#2(human)	Thermo Fisher Scientific	s41367	human STX1B	CGGUCCAAAUUGAAAGCGA
siSTX4#1(human)	Thermo Fisher Scientific	STX4HSS110344	human STX4	UGUCCCAGCAAUUCGUGGAGCUCAU
siSTX4#2(human)	Thermo Fisher Scientific	STX4HSS186154	human STX4	AGGUCCGGACAAUUCGGCAGACUAU
siVAMP5(dog)	Nippon gene		dog VAMP5	UUGGCCUGCUCAUCAUCCU
siSNAP47(dog)	Nippon gene		dog SNAP47	UGCAUCUCAUGUUAAAGCA
siSTX1A(dog)	Nippon gene		dog STX1A	UCCGAGGCUUCAUUGACAA
siSTX1B(dog)	Nippon gene		dog STX1B	GCUGUGAAAUAUCAGAGCA
siSTX4(dog)	Nippon gene		dog STX4	UUGUCAAGCUGGAGAAUAA
